# Tachycardia-Induced Cardiomyopathy in a Young Adult: The Significance of Early Diagnosis and Treatment

**DOI:** 10.7759/cureus.35531

**Published:** 2023-02-27

**Authors:** Oluwaremilekun Tolu-Akinnawo, Oghanim I Ogwu, Akintomiwa Akintunde, Nkechi Akata, Henry Okafor

**Affiliations:** 1 Internal Medicine, Meharry Medical College, Nashville, USA; 2 Cardiology, Vanderbilt University Medical Center, Nashville, USA

**Keywords:** tachycardia-induced cardiomyopathy, early identification and diagnosis, reversible cardiomyopathy, young adult, cardiomyopathy, tachycardia

## Abstract

Tachycardia-induced cardiomyopathy (TIC) is gradually gaining the attention it deserves as one of the most common causes of reversible cardiomyopathy. Although TIC appears common, there has been limited data, especially among young adults. Patients with tachycardia and left ventricular dysfunction should be suspected of having TIC, with or without established etiology of heart failure, because TIC can develop by itself or contribute to cardiac dysfunction.

We present a case of a previously healthy 31-year-old woman with persistent nausea and vomiting, poor oral intake, fatigue, and persistent palpitations. Vital signs at presentation were significant for tachycardia of 124 beats per minute, which she reported was similar to her baseline heart rate of 120s per minute. There were no apparent signs of volume overload at the presentation. Labs were significant for microcytic anemia with hemoglobin/hematocrit of 10.1/34.4 g/dL, and mean corpuscular volume was low at 69.4 fL; other labs were unremarkable. Transthoracic echocardiography obtained at admission was significant for mild global left ventricular hypokinesis, systolic dysfunction with an estimated left ventricular ejection fraction of 45-50%, and mild tricuspid regurgitation. Persistent tachycardia was suggested as the primary cause of cardiac dysfunction. The patient was subsequently started on guideline-directed medical therapy, including beta blockers, angiotensin-converting enzyme inhibitors, and spironolactone, with eventual normalization of the heart rate. Anemia too was also treated. Follow-up transthoracic echocardiography done four weeks after was notable for significant interval improvement in left ventricular ejection fraction of 55-60%, with a heart rate of 82 beats per minute.

The case illustrates the need for early identification of TIC regardless of the patient’s age. It is essential that physicians consider it in the differential diagnosis of new-onset heart failure because prompt treatment leads to the resolution of symptoms and improvement of ventricular function.

## Introduction

Long-standing tachycardia is a well-established cause of left ventricular systolic dysfunction [1]. Tachycardia-induced cardiomyopathy (TIC) is defined as systolic and/or diastolic ventricular dysfunction secondary to prolonged elevated heart rate, reversible upon control of the arrhythmia or heart rate. The first TIC case was identified by Gossage and Hicks in 1913 when a young male patient presented with clinical features of congestive heart failure and atrial fibrillation with rapid ventricular response [2]. In 1949, Phillips and Levine described an association between atrial fibrillation and reversible cardiomyopathy [3]. However, the first experimental model was first described by Whipple et al. through an experimental model on ventricular systolic dysfunction in 1962 [4]. This model has been used as a foundation for further studies on this topic. TIC is one of the major causes of congestive heart failure, a significant health concern with a high burden of morbidity and mortality [5]. The appropriate management of tachycardia and heart failure can dramatically improve TIC. Therefore, early recognition of TIC is essential. In this review, we will discuss the case presentation, diagnostic strategy, treatment of TIC, and the role of active surveillance in the early resolution of TIC.

## Case presentation

A 31-year-old female presented to the emergency department with a six-week history of unintentional weight loss of about 30 pounds, progressive fatigue, and persistent palpitations. Symptoms initially started about three months earlier with epigastric pain, persistent nausea, vomiting, and associated poor oral intake. She also reported dizziness and progressive fatigue, necessitating presentation at an outside facility. An upper endoscopy was significant for gastritis, and she was placed on a proton pump inhibitor. However, with persistent fatigue and palpitations, the patient decided to present to our facility. Of note, she had no previous history of cardiac disease.

On physical examination, her height was 64 inches, weight was 54 kg, blood pressure was elevated at 129/94 mmHg, heart rate was elevated at 124 per minute, which she reported was similar to her baseline heart rate at 120s per minute, respiratory rate was 18 per minute, and body temperature was 98.8°F. Jugular venous distention was absent, and no cervical and axial lymphadenopathy was noted. The heart sounds were tachycardic; however, regular with a presystolic gallop (S4). The breath sounds were clear, without rales or wheezing. There was no definite pitting edema or tremors. Otherwise, the physical findings were unremarkable. The patient denied the use of illicit substances.

Lab findings at admission were significant for hemoglobin/hematocrit low at 10.1/34.4 g/dL (reference: 12.0-16.0 g/dL), mean corpuscular volume (MCV) low at 69.4 fL (reference: 80.0-99.0 fL), white cell count of 8.8 g/dL (reference: 4.5-10.0 g/dL), D-dimer elevated at 2.12 mg/L (reference: 0.4-2.0 mg/L), and sodium at 138 mmol/L (reference: 136-145 mmol/L). Liver function and thyroid function tests were within normal limits. Troponins and pro-B-type natriuretic peptides were within normal limits. Urinalysis showed 2+ protein and was otherwise unremarkable. Coronavirus disease 2019 (COVID-19) testing was negative. The urine drug screen was negative.

The chest radiography film revealed cardiomegaly and was otherwise unremarkable. Electrocardiography (ECG) showed sinus tachycardia with premature atrial complexes and non-specific T-wave abnormalities.

**Figure 1:**
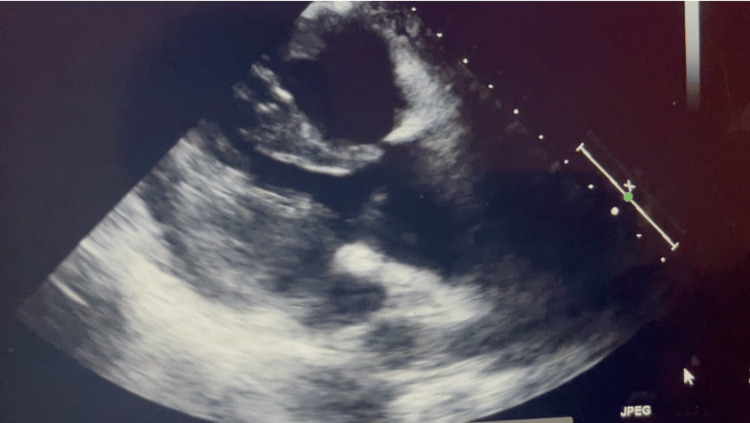
Two-dimensional echocardiography with an estimated left ventricular ejection fraction of 45-50% and grade 1 diastolic dysfunction, with aortic root dilation at 4 cm

The echocardiography (ECHO) revealed mild global left ventricular (LV) hypokinesis and systolic dysfunction with an estimated LV ejection fraction of 45-50%, grade 1 diastolic dysfunction, with aortic root dilation at 4 cm, and mild tricuspid regurgitation (Figure 1).

Pulmonary CTA was negative for pulmonary embolism. CT of the abdomen and pelvis was negative for adrenal mass.

**Figure 2:**
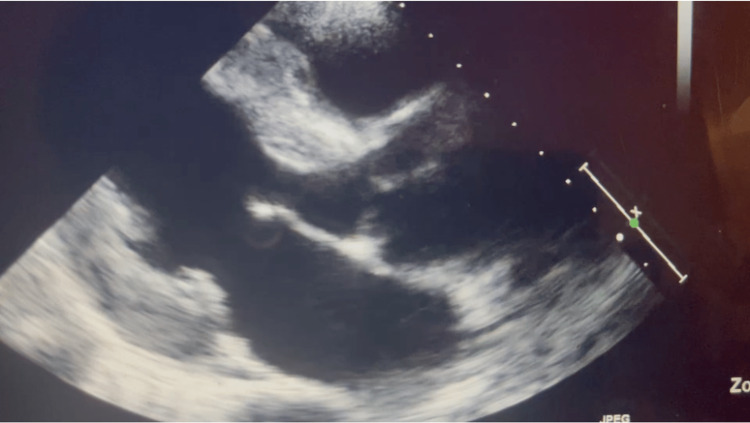
Repeat echocardiography noted interval improvement of systolic function with a left ventricular ejection fraction of 55-60%

The presentation was clinically consistent with persistent tachycardia with combined systolic and diastolic heart failure. The patient was conservatively treated with guideline-directed medical therapy, including a beta blocker, and was discharged to follow up with a primary care provider (PCP) and cardiologist on an outpatient basis. Follow-up at the cardiology clinic four weeks later was notable for regular heart rate, and repeat ECHO noted interval improvement of systolic function with LV ejection fraction of 55-60% (Figure 2).

Follow-up visits

This patient's first suspicion of TIC was during the present admission. Early identification and commencement of rate control medication were vital to the improvement in cardiac function.

At the follow-up visit at the PCP office, a beta-blocker dosage adjustment was made. The patient also continued guideline-directed medical therapy with angiotensin-converting enzyme inhibitors/spironolactone as the blood pressure permitted.

At the follow-up with the cardiologist, Holter monitoring was significant for a minimum heart rate of 31 beats per minute, a maximum heart rate of 222 beats per minute, and an average heart rate of 92 beats per minute while on a beta blocker, with non-sustained ventricular tachycardia (NSVT) detected within +/- 45 seconds of the symptomatic patient event(s). A repeat transthoracic ECHO revealed an interval improvement in ventricular function with a low normal estimated LV ejection fraction of 55-60%. The patient also underwent a nuclear medicine stress test, which was negative for ischemia, and an elective left heart catheterization, which was negative for obstructive coronary artery disease.

## Discussion

Pathophysiology

In this case, we suspect a combined systolic and diastolic cardiac dysfunction related to persistent tachycardia. Clinical studies have found a variable time from the onset of tachyarrhythmia to the development of TIC, ranging from three to 120 days [5]. The heart rate limit for which TIC could occur is yet to be established; however, a prolonged heart rate greater than 100 beats per minute should raise concerns and be controlled [6]. Abnormally increased ventricular rates initially result in cardiac dilatation and mitral regurgitation, which in turn are usually associated with elevated ventricular filling pressures, decreased contractility, ventricular wall thinning, and eventually heart failure with neurohormonal activation [6]. In experimental models, some of these changes are identified as early as 24-48 hours after rapid cardiac pacing, with continued deterioration in ventricular function noted for up to three to five weeks. A study revealed a shorter time lag between the diagnosis of tachycardia and TIC in older patients compared to younger patients [7]. Although the pathway is not entirely clear currently, proposed mechanisms involve cellular changes, including myocyte loss, cellular elongation, myofibril misalignment, and loss of sarcomere register [5]. All of these have been noted to lead to the derangement of the extracellular matrix.

Management and cardiac recovery

Also, it is important to note that because TIC is rate dependent, the higher the heart rate, the higher the risk of TIC development and progression [8,9]. Normalization of heart rate, either through rate or rhythm control, remains the mainstay of treatment of TIC, with most data coming from patients with atrial fibrillation [6]. In other clinical scenarios, termination of the underlying arrhythmia using antiarrhythmic agents such as amiodarone and direct current cardioversion may be needed [6]. For refractory cases, a catheter ablation such as radio frequency catheter ablation therapy can be a definitive cure [6]. Also, treating the underlying cause of tachycardia is as significant. Previous studies have also documented improvement in cardiac output and cardiopulmonary exercise testing variables of ventricular rate control, maximal oxygen uptake, and anaerobic threshold after cardioversion of atrial fibrillation back to sinus rhythm in cardiomyopathy patients [4]. However, complete recovery of ventricular function is rare. Also, the duration of ventricular dysfunction recovery in a patient with TIC varies; however, both experimental and human studies have shown the greatest left ventricular ejection fraction improvement within one month after arrhythmia resolution or control of heart rate, just as in our patient [10-13]. Recovery of LV ejection fraction usually does not take more than six months if the tachycardia culprit is well controlled [4].

Role of early suspicion and active surveillance

In our patient’s case, the timely initiation of rate control medication helped with interval improvement in cardiac function within the one-month follow-up. Also, active treatment of the underlying cause and active surveillance through follow-up visits with the primary care provider and cardiology clinics helped with medication adjustments and monitoring of cardiac function. Early control of tachycardia has been associated with improved survival in TIC, hence, the role of awareness in the management of TIC [4]. Physicians, primarily primary care providers, should always suspect tachycardia as a possible cause of cardiac dysfunction, especially when there is no apparent explanation for cardiomyopathy. They also should endeavor to treat the underlying cause of tachycardia, as this is equally important.

It is also important to note that patients who recovered from TIC are at an increased risk of recurrence. In these cases, the left ventricular systolic dysfunction is usually more rapid and severe than in previous TIC events [14,15]. This suggests that, although the left ventricular ejection fraction normalizes, some structural abnormalities majorly related to the remodeling process persist. It is also not uncommon to have sudden cardiac death even after appropriate treatment and clinical improvement in TIC [14,15]. This emphasizes the need for active surveillance despite improvement.

## Conclusions

In conclusion, we present a case of TIC, which significantly improved with rate control. Early suspicion and treatment are crucial and associated with long-term benefits in most patients. Awareness is vital as it helps with early detection and management. Physicians need to consider TIC as a differential diagnosis in patients of any age who present with persistent tachycardia and heart failure because prompt treatment resolves symptoms and improves ventricular function. There are limited data on the etiologies of TIC in young adults. Hence, future research should focus on this as the burden is gradually increasing. Also, there is a need for further studies on genetic and clinical characteristics guiding risk predictions of TIC, which will help with the early detection of high-risk patients and treatment with the goal of preventing heart failure.
